# Visible fluid motion on manipulation as the new threshold for intraoperatively determined knee arthroplasty component loosening: A Delphi study

**DOI:** 10.1002/ksa.12357

**Published:** 2024-07-15

**Authors:** George S. Buijs, Arthur J. Kievit, Alex B. Walinga, Matthias U. Schafroth, Michael T. Hirschmann, Leendert Blankevoort, International Consensus Panel

**Affiliations:** ^1^ Department of Orthopedic Surgery and Sport Medicine Amsterdam UMC, Location AMC Amsterdam The Netherlands; ^2^ Amsterdam Movement Sciences, Musculoskeletal Health Amsterdam The Netherlands; ^3^ Department of Orthopedic Surgery and Traumatology Kantonsspital Baselland Bruderholz Switzerland; ^4^ Department of Clinical Research Research Group Michael T. Hirschmann, Regenerative Medicine & Biomechanics, University of Basel Basel Switzerland

**Keywords:** aseptic loosening, consensus, diagnostic accuracy, intraoperative findings, knee arthroplasty, reference test, revision knee arthroplasty

## Abstract

**Purpose:**

There is a lack of a clear, uniform definition for intraoperatively assessed component loosening of a knee arthroplasty component, complicating the interpretation and interchangeability of results of diagnostic studies using an intraoperative observation as the reference test. The purpose of this study was to establish a consensus among specialised knee revision surgeons regarding the definition of intraoperatively determined loosening of total or unicondylar knee arthroplasty components.

**Methods:**

Utilising the Delphi consensus method, an international panel of highly specialised knee revision surgeons was invited to participate in a three‐round process. The initiation of the first round involved the exploration of possible criteria for intraoperatively determined loosening with open questions. The second round focused on rating these criteria importance on a five‐point Likert scale. For the third round, criteria that reached consensus were summarised in consecutive definitions for intraoperatively determined loosening and proposed to the panel. Consensus was established when over 70% of participants agreed with a definition for intraoperatively determined loosening.

**Results:**

The 34 responding panel members described in total 60 different criteria in the first round of which 34 criteria received consensus in the second round. Summarising these criteria resulted in four different definitions as minimal requirements for intraoperatively determined loosening. Eighty‐eight percent of the panel members agreed on defining a component as loose if there is visible fluid motion at the interface observed during specific movements or when gently applying direct force.

**Conclusion:**

This study successfully established a consensus using a Delphi method among knee revision surgeons on the definition of intraoperatively determined component loosening. By agreeing on the visibility of fluid motion as new definition, this study provides a standardised reference for future diagnostic research. This definition will enhance the interpretability and interchangeability of future diagnostic studies evaluating knee arthroplasty component loosening.

**Level of Evidence:**

Level V.

**Abbreviations**
rTKA: revision total knee arthroplasty
TKA: total knee arthroplasty
UKA: unicondylar knee arthroplasty

## INTRODUCTION

Following total knee arthroplasty (TKA) and unicondylar knee arthroplasty (UKA), the likelihood for revision TKA (rTKA) within a decade is 13% for TKA and 12% for UKA [8, 17]. Given the rise in numbers of primary TKA and UKA, the incidence of revision surgeries is expected to rise, despite advancements in implant design and surgical techniques [15, 16, 18].

Aseptic loosening accounts for 20%–30% of these rTKAs, making it the most prevalent reason for TKA failure [6, 21]. Aseptic TKA or UKA loosening often necessitates complex and demanding revision surgery, presenting substantial challenges to both patients and global healthcare infrastructure [12, 13]. Correct diagnosis is essential to avoid unnecessary revision surgery in patients misdiagnosed with loosening of the TKA, and to ensure that those with undetected loosening receive the required revision interventions. It also eliminates unnecessary delays in providing patients with the proper care based on an accurate diagnosis.

Efforts to refine the diagnostic process for aseptic loosening have led to a multitude of research exploring different diagnostic approaches and modalities [1, 2, 3, 14, 22, 23]. These studies often reported conflicting outcomes, contributing to a considerable degree of inconsistency and variability in the importance and value attributed to the results of various available diagnostic modalities [3, 4]. These conflicting results stem largely from a high risk of bias concerning the reference test employed in these studies. In the absence of tools to quantify implant motion, most diagnostic studies employ intraoperative visual assessment of component loosening as the main reference test [1, 3, 5]. A recent systematic review assessed fourteen diagnostic studies evaluating modalities utilised to assist in diagnosing aseptic knee arthroplasty component loosening [5]. Only three of the included studies clearly defined the method by which intraoperative assessment of component loosening was dichotomised as either loose or fixed. Among these, two disparate criteria were identified. One study employed the ability to remove a component with one hand as threshold for component loosening as opposed to the other study were the ability to toggle the implant was deemed sufficient to conclude loosening [5, 9, 19]. The divergence in definitions for loosening across these studies may have contributed to disparate results when applied interchangeably. This supposition is assisted by the high variability in the confidence attributed to various available diagnostic modalities, as reported in a Delphi consensus study resulting from the same Delphi project as the present study [4].

Consequently, the orthopaedic community continues to encounter difficulties in formulating a uniform diagnostic standard for aseptic loosening, crucial for improving treatment selection and enhancing outcomes for patients [25].

Amidst these uncertainties, no study has been conducted to evaluate the variation of opinions and to define consensus regarding the definition of an intraoperatively determined loose TKA or UKA component among knee revision specialists. There is need for a clearer clinical guideline regarding aseptic loosening to avoid both overtreatment and undertreatment. In the absence of any consensus based on literature data, a Delphi study might help to arrive at a consensus on a threshold for component loosening for future use in diagnostic accuracy test studies for aseptic knee arthroplasty loosening. This study aims to answer the following two research questions using a Delphi Consensus method:

(1) Is there consensus among highly specialised knee revision surgeons regarding the definition of intraoperatively determined loosening of TKA or UKA components?
(2) What is the extent of variability among highly specialised knee revision surgeons when a TKA or UKA component should be considered loose when intraoperatively determined?

It was hypothesised that consensus could be reached, while high variability would be observed.

## MATERIALS AND METHODS

The methods employed in the present Delphi consensus article are similar to those employed for a recent Delphi consensus article evaluating variability and consensus between diagnostic modalities used to diagnose aseptic knee arthroplasty loosening [4]. For this Delphi consensus project, the reporting guidelines and methodological standards as recommended by Jünger et al. and Diamond et al. were adopted and an international consensus panel of highly specialised knee revision surgeons was established [7, 11]. The current Delphi study unfolded over three different rounds. The first round focused on identifying all opinions on criteria for intraoperative loosening according to panel members. The second round involved rating the importance of these identified criteria. In case uniform consensus has not been achieved by the end of the second round, a third round was prepared to reassess the criteria, informed by a summary of the second round's results. This study focused exclusively on the loosening of tibial and femoral components.

The lead author (G. S. B.) with help of a co‐author (A. B. W.) took on the role of coordinator, formulating the questionnaires based on feedback from participants and overseeing communications. To eliminate the risk of moderator bias affecting the study's results, G.S.B. and A. B. W. abstained from joining the study as a panellist. Although co‐authors A. J. K., M. U. S. and M. T. H. were panel members, they were excluded from the response processing and analysis phase to ensure impartiality.

### Assembling the international consensus panel

Three different methods were used to identify orthopaedic surgeons with expertise in knee arthroplasty revision: (1) by reviewing the authorship of high‐quality articles focused on diagnosing aseptic loosening in knee arthroplasty, (2) by examining the programmes of orthopaedic conferences to identify keynote speakers discussing or presenting on topics related to knee arthroplasty and loosening and (3) via the professional networks of the co‐authors. This strategy was implemented to guarantee a panel of participants that was varied, international and representative.

Currently, no definitive guidelines or established benchmarks for the optimal number of participants in Delphi studies are available. While some researchers argue that a cohort of 10–15 members might be adequate for more uniform groups, a larger sample is often recommended when a larger variety is expected [10, 24]. For this Delphi consensus study, an a priori minimum of 25 panel members was required. In anticipation of potential nonresponders, 69 candidates were identified and invited as potential panel members. Those who agreed to complete participation were sent the Delphi consensus questionnaires.

### Baseline

First, participants were requested to disclose their years of experience in knee revision surgery and the annual average number of knee arthroplasty revision surgeries. Additionally, information regarding their gender and the country of their current practice were collected. Participants also had the option to indicate if they wished to be recognised for participation as a panel member. Such acknowledgement as a group author was contingent upon a participant completing all rounds of the consensus process.

### First round

To gain a better understanding of the different opinions and standpoints towards the issue of intraoperative assessment of component loosening between the participants, the following two questions were asked:

(1) *To what extent do you agree that there is no clear uniform definition in available literature for what is to be defined as loose and fixed in intraoperative assessment (during revision surgery) of knee arthroplasty component loosening?*
(2) *To what extent do you agree that there is a need for a uniform definition of what is to be defined as loose and fixed in intraoperative assessment (during revision surgery) of knee arthroplasty component loosening?*

Both questions were Single Select Multiple Choice Questions using a 5‐point Likert scale (1: *I fully agree*, 2: *I mostly agree*, 3 *neutral*, 4; *I do not agree completely* and 5; *I do not agree at all*).

The first round identified criteria associated with intraoperative assessment of loosening of both the tibial and femoral component of a TKA, UKA or rTKA, separately. Therefore, two questions were posed:

(1) *How would you personally during revision knee arthroplasty define loosening of the tibia component? (Please answer separately for partial, primary and revision surgery)*.
(2) *How would you personally during revision knee arthroplasty define loosening of the femur component? (Please answer separately for partial, primary and revision surgery)*.

### Second round and third round

Ahead of the second round, all statements were collected, and any duplicates were removed. All statements were incorporated in the second‐round survey *as:*

‘“statement” should result in the judgement that the *tibial* component of a (either partial, primary or revision) knee arthroplasty is loose’.

and as:

‘“statement” should result in the judgement that the *femoral* component of a (either partial, primary or revision) knee arthroplasty is loose’.

Since no unique differences were noted in the first round, all statements were deemed relevant to UKA, TKA and/or rTKA in the second round. Panel members were then requested to express their level of agreement with these statements using a 5‐point Likert scale.

The outcomes of the second round were compiled into frequency tables and shared with all participants. High variability regarding a particular statement was defined as the statement having received at least 1 score of complete disagreement and at least 1 score of complete agreement, both must apply.

Statements meeting the consensus level (>70% fully agree or mostly agree) were summarised into different and distinctive definitions for a loose component and presented as consecutive minimal requirements ranked based on the degree of severity of looseness and thus expected mobility of the component. Participants were asked to point out their minimal definition for a component to be considered as loose. It was made clear that by pointing out the minimal definition, all definitions that testify to a larger degree of expected mobility were also interpreted as fitting the definition of a loose component.

Consensus was established when over 70% of participants deemed a proposed definition as fitting the definition of a loose component.

### Data collection and analysis

Baseline demographic information was reported using mean and standard deviation or median and interquartile range, depending on the distribution of the data. The categorical variable ‘consensus’ was depicted in terms of absolute numbers and percentages. Participants who did not complete the first round were disqualified from subsequent rounds and their data were not included in the analysis. Data were analysed using Excel 16.0 (Microsoft Excel 2016; Microsoft Corp.). Questionnaires were distributed using Castor EDC (2023.4.0.0).

## RESULTS

### Panel characteristics

**Figure 1:**
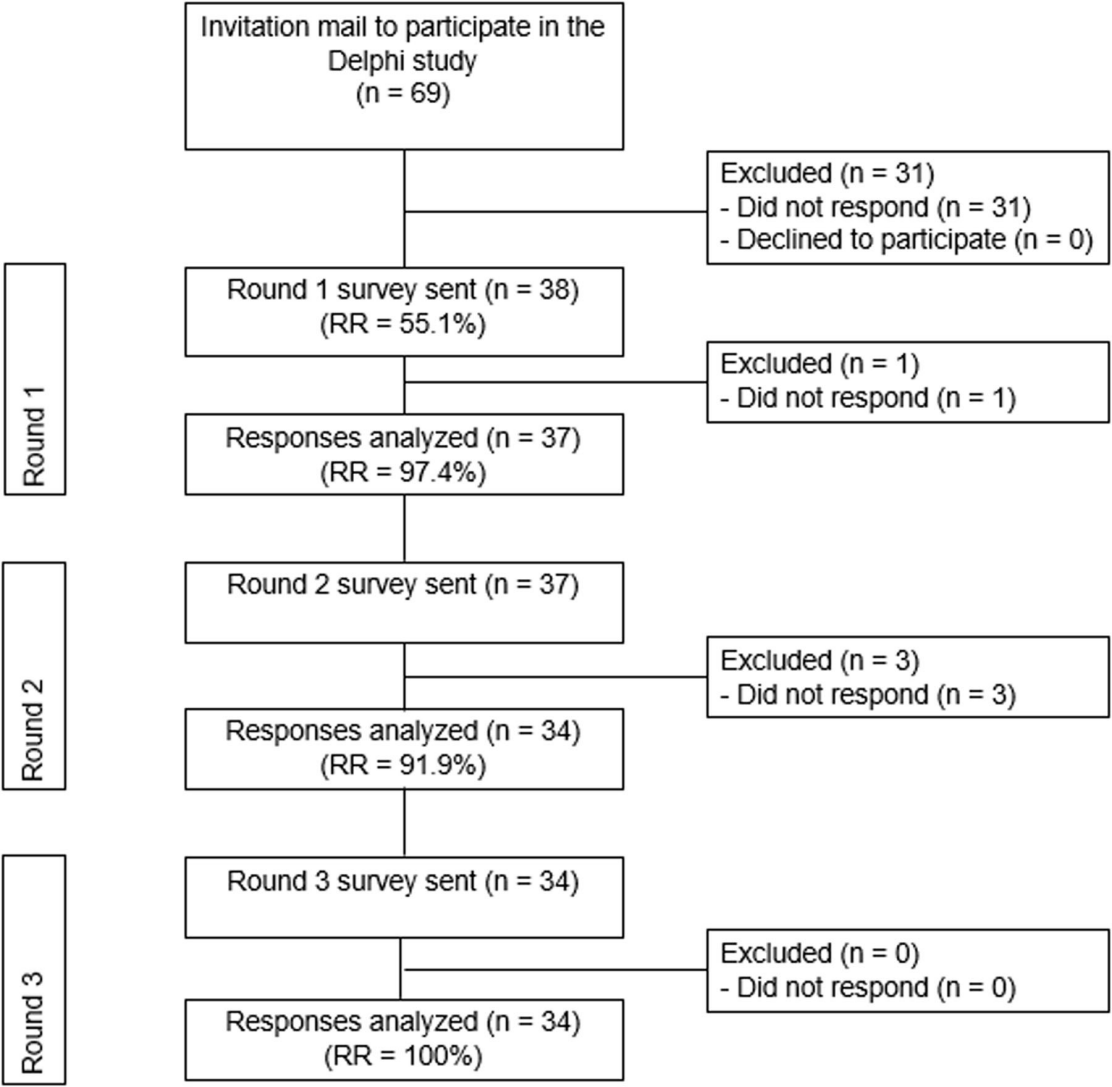
Flowchart of inclusion of panel members and response rates (RR) per round.

A total of 69 eligible panel members were contacted by email and 38 (55.1%) agreed to participate in this Delphi consensus study, scheduled from September 2023 to December 2023. All but one (*n* = 37; 97.4%) who agreed to participate completed the first round. Three participants (8.1%) failed to complete the second round. All participants (100%) included in the second round completed the third round. Therefore, all rounds were completed by a total of 34 (89.5%) of the 38 originally included participants (Figure 1).

The Dutch panel members formed the predominant group, comprising 26.5% (*n* = 9) of all participants (Table 1). The majority of participants were male (*n* = 32; 94.1%; Table 1). Among the 34 participants, 31 (91.2%) conducted more than 20 knee arthroplasty revision surgeries annually. Most participants (*n* = 28; 82.4%) had more than 10 years of experience as knee arthroplasty revision surgeons (Table 1).

**Table 1:** Baseline characteristics of participants completing all rounds of this Delphi consensus study.

Characteristics	*n*	Percentages
Gender		
Male	32	94.1
Female	2	5.9
Country/region of practice		
The Netherlands	9	26.5
United States	4	11.8
Germany	4	11.8
United Kingdom	4	11.8
Belgium	3	8.8
Switzerland	2	5.8
France	2	5.8
India	2	5.8
Indonesia	1	2.9
Peru	1	2.9
Türkiye	1	2.9
Spain	1	2.9
Experience as knee arthroplasty revision surgeon (years)		
0−5	2	5.9
5−10	4	11.8
10−15	12	35.2
20−25	9	26.4
>25	7	20.6
Average knee arthroplasty revision surgeries per year		
0−10	1	2.9
10−20	2	5.9
20−30	9	26.5
30−40	4	11.8
40−50	6	17.7
>50	12	35.3

### First round

Thirty‐seven panel members completed the first round. Between these 37 members, 31 (83.8%) agreed fully or mostly with the statement that there is no clear uniform definition of knee arthroplasty component loosening when determined intraoperatively. The majority (*n* = 34; 91.9%) either fully or mostly agreed with the statement that there is a need for a clear uniform definition for intraoperatively determined knee arthroplasty component loosening.

All members in the first round reported a total of 28 distinctive statements regarding the UKA tibial component, 29 statements concerning the TKA tibial component and 32 statements concerning the rTKA tibial component. Regarding the femoral component, 27 statements concerning UKA, 28 statements concerning primary TKA and 31 statements concerning rTKA were proposed. Distinctions between cemented and uncemented components were made by the participants. Very few technically relevant differences were found between statements regarding either UKA, primary TKA or rTKA. Therefore, discrimination between different types of knee arthroplasties was not further applied, unless specified otherwise.

### Second round

Thirty‐four participants completed the second round. In preparation for the second round, all statements were summarised as separate statements for either the tibial or femoral component. This resulted in 31 unique statements regarding the tibial component and 29 unique statements regarding the femoral components, of which ‘Pedestal formation around the tip of the stem’ and ‘Perforation of a stem through the cortex’ were solely applicable to suspected loosening of an rTKA component. Ten proposed statements were applicable to cemented components. Six proposed statements were applicable to uncemented components. Forty‐four statements were applicable to both cemented and uncemented components. The applicability of a statement to cemented components, uncemented components or both were specified in the summary.

A total of 34 statements received >70% consensus, of whom 18 applicable to the tibial component and 16 applicable to the femoral component (Tables 2a and 2b).

**Table 2a:** Level of agreement with statements concerning tibial components.

Statement	Percentages of agreement (fully and mostly agreed)
Cemented: Complete detachment of bone–cement interface or cement–prosthesis interface, resulting in easy (without force/osteotome) removal of prosthesis with clamp	100^a^
Uncemented: Complete detachment of prosthesis–bone interface with interposition of fibrous tissue, resulting in easy (without force/osteotome) removal of prosthesis with clamp	100^a^
Both: lifting of the anterior portion of the prosthesis in deep flexion	97.1^a^
Both: Macroscopic mobility of the implant during varus or valgus stress	94.1^a^
Both: Macroscopic mobility of the implant with applied force by finger	94.1^a^
Both: Macroscopic mobility of the implant during flexion or extension	91.2^a^
Both: Macroscopic mobility of the implant independent of method force was applied	91.2^a^,^b^
Both: Macroscopic mobility of the implant when pushing implant to bone after single impaction	91.2^a^
Both: Migrated component	91.2^a^
Both: Macroscopic mobility of the implant when tested with probe or osteotome	88.2^a^
Both: Visible fluid motion beneath component when gently lifting	88.2^a^,^b^
Cemented: Visual subsidence of component	88.2^a^
Both: Ability to tap‐off the component with minimal effort	82.4^a^
Cemented: Visual failure of bone–cement interface	79.4^a^
Both: Knibbling away anterior bony overgrowth, then stress the component in 10° of flexion with varus and valgus stress and watch for fluid or bubbles going in and out the interface region	73.5^a^
Both: Macroscopic mobility of the implant with applied force by ‘punch’ of hammer	73.5^a^,^b^
Cemented: No cement fixation on component	70.6^a^,^b^
Uncemented: Lack of osteointegration of the component	70.6^a^
Both: Release of the component from bone or cement after few strokes with osteotome	64.7
Both: Easy to extract	58.8
Both: Fibrous tissue between implant–bone interface or implant–cement interface	55.9
Uncemented: Clear gap between component and bone	55.9
Both: Easy removal with Chisel	52.9
Both: Clear area of granulation at interface of bone	50.0
Both: presence of a depressed plateau	44.1
Both: After removal; presence of zones of bone resorption around the placement of the component	38.2^b^
Both: Pedestal formation around the tip of the stem (for revision only)	38.2^b^
Both: Perforation of a stem through the cortex (for revision only)	38.2^b^
Both: Mispositioned component	11.8^b^
Cemented: Visual cement particles	11.8^b^
Both: Intraosseous antibiotic penetrating directly into the joint space from a tibial injection	2.9

*Note*: Both: statement applies to both cemented and uncemented components.

a The preset threshold for consensus was met.

^b^ The preset threshold for high variability were met.

**Table 2b:** Level of agreement with statements concerning femoral components.

Statement	Percentages of agreement fully and mostly agreed)
Cemented: Complete detachment of bone–cement interface or cement–prosthesis interface, resulting in easy (without force/osteotome) removal of prosthesis with clamp	100^a^
Uncemented: Complete detachment of prosthesis–bone interface with interposition of fibrous tissue, resulting in easy (without force/osteotome) removal of prosthesis with clamp	100^a^
Both: Macroscopic mobility of the implant when tested with probe or osteotome	94.1^a^
Both: Macroscopic mobility of the implant during flexion or extension	94.1^a^
Both: Macroscopic mobility of the implant with applied force by finger	94.1^a^
Both: Macroscopic mobility of the implant independent of method force was applied	91.2^a^
Both: Macroscopic mobility of the implant during varus or valgus stress	91.2^a^
Both: lifting of the anterior portion of the prosthesis in deep flexion	91.2^a^
Both: Migrated component	91.2^a^
Cemented: Visual subsidence of component	85.3^a^
Both: Ability to tap‐off the component with minimal effort	85.3^a^
Both: Macroscopic mobility of the implant when pushing implant to bone after single impaction	82.4^a^
Cemented: Visual failure of bone–cement interface	82.4^a^
Both: Macroscopic mobility of the implant with applied force by ‘punch’ of hammer	79.4^a^
Uncemented: Lack of osteointegration of the component	76.5^a^, b
Cemented: No cement fixation on component	73.5^a^,^b^
Both: Visible fluid motion beneath component when gently lifting	67.6^b^
Both: Knibbling away anterior bony overgrowth, then stress the component in 10 ^o^ of flexion with varus and valgus stress and watch for fluid or bubbles going in and out the interface region	64.7
Both: Release of the component from bone or cement after few strokes with osteotome	61.8
Both: Easy removal with Chisel	61.8^b^
Both: Easy to extract	58.8^b^
Both: Clear area of granulation at interface of bone	50.0^a^
Uncemented: Clear gap between component and bone	50.0^b^
Both: Perforation of a stem through the cortex (revision only)	47.1^b^
Both: After removal; presence of zones of bone resorption around the placement of the component	44.1^b^
Both: Pedestal formation around the tip of the stem (revision only)	41.2^b^
Both: Mispositioned component	20.6^b^
Cemented: Visual cement particles	17.6^b^
Both: Intraosseous antibiotic penetrating directly into the joint space from a tibial injection	11.8^b^

*Note*: Both: statement applies to both cemented and uncemented components.

a The preset threshold for consensus was met.

^b^ The preset threshold for high variability were met.

High variability was observed in 22 (64.7%) statements, of whom nine were applicable to tibial components and 13 applicable to femoral components (Tables 2a and 2b). Between those 22 statements, eight statements were identical between statements concerning tibial and femoral components.

Between the statements that received consensus, high variability was observed in three statements applicable to the tibial component and two statements to the femoral component. For the tibial component, these were ‘Both: Macroscopic mobility of the implant with applied force by ‘punch’ of hammer’, ‘Both: Visible fluid motion beneath component when gently lifting’ and ‘Cemented: No cement fixation on component’. For the femoral components, these were ‘Cemented: No cement fixation on component’ and ‘Cementless: Lack of osteointegration of the component’.

### Third round

Thirty‐four participants completed the third round. In preparation for the third round, all 34 statements receiving consensus were summarised and duplicates between those applicable to both tibial and femoral components were removed. As very few technically relevant differences were found between statements regarding tibial or femoral components, discrimination between the two different components was not further applied. This resulted in the four consecutive definitions of component loosening that were presented together with the following explanation (Table 3).

**Table 3:** Levels of agreement with proposed definitions for intraoperatively determined loosening, with scores, cumulative scores and percentages of cumulative scores for each proposed definition of intraoperatively determined loosening.

#	Definition	Count of times pointed out	Cumulative agreement	Agreement (%)
1	A tibial and/or femoral component is considered loose if there is complete detachment at the interface, which is indicated by the easy removal of the prosthesis without the need for additional force or tools. This applies to both cemented implants (bone–cement or cement–prosthesis interface) and uncemented implants (prosthesis–bone interface).	4	4	11.8
2	A tibial and/or femoral component is considered loose if there is macroscopic mobility of the implant observed during specific movements such as flexion, extension, varus or valgus stress or when applying direct gentle force with a finger or instrument (e.g., probe or osteotome). This applies to both cemented implants (bone–cement or cement–prosthesis interface) and uncemented implants (prosthesis‐bone).	11	15	44.1
3	A tibial and/or femoral component is considered loose if there is visible fluid motion at the interface (without macroscopic mobility of the implant) observed during specific movements such as flexion, extension, varus or valgus stress or when gently applying direct force with a finger or instrument (e.g., probe or osteotome). This applies to both cemented implants (bone–cement or cement–prosthesis interface) and uncemented implants (prosthesis–bone).	15	30	88.2
4	A tibial and/or femoral component is considered loose if there is macroscopic mobility of the implant after single impaction (e.g., with a hammer). This applies to both cemented implants (bone–cement or cement–prosthesis interface) and uncemented implants (prosthesis–bone).	4	34	100

‘Based on the results of the second round, four statements are formulated, ranked by the degree of severity of the looseness and expected mobility of the component. In your opinion, which of these four statements is the minimum requirement for a component to be considered loose?’

With agreement of 88.2% of the panel members, the preset level of agreement was met for the following definition for intraoperatively determined loosening;

**Table:** 

(3) A tibial and/or femoral component is considered loose if there is visible fluid motion at the interface (without macroscopic mobility of the implant) observed during specific movements such as flexion, extension, varus or valgus stress or when gently applying direct force with a finger or instrument (e.g., probe or osteotome). This applies to both cemented implants (bone–cement or cement–prosthesis interface) and uncemented implants (prosthesis–bone).

Results of the third round are shown in Table 3.

## DISCUSSION

The most important finding of the present study was the consensus on the visibility of fluid motion at the interface between the TKA component and/or cement and the bone observed during specific movements, such as flexion, extension, varus or valgus stress or when gently applying direct force with a finger or instrument, as the definition for intraoperatively determined knee arthroplasty component loosening. This threshold should be applied when using intraoperatively determined loosening as a reference test in future diagnostic accuracy test studies. This study also revealed considerable variance in expert views, with high variability observed in 64.7% of the statements posed in the second round (Tables 2a and 2b).

These results emphasise the variability in standards applied when testing for TKA or UKA component loosening intraoperatively, yet they also underscore a general agreement among international specialised knee revision surgeons. This resulted in a new definition for intraoperatively determined TKA or UKA component loosening. This new definition will standardise the use of intraoperatively testing of component loosening as reference test in future diagnostic research. Given the crucial role of accurate diagnosis, this new definition will help reduce incomparability between the reported diagnostic accuracy of modalities used to aid the diagnosis of aseptic knee arthroplasty loosening and, therefore, will help reduce both unnecessary and uncommitted but necessary revision surgeries.

This study is the first to evaluate variability and establish consensus for intraoperatively determined knee arthroplasty component loosening. The need for consensus was identified after conducting a systematic review and meta‐analysis. This systematic review evaluated fourteen studies on diagnostic methods for identifying aseptic loosening in knee arthroplasty components. Only three studies provided a clear methodology for distinguishing between loose and fixed components during surgery, revealing inconsistencies with two differing criteria identified. According to Mayer‐Wagner et al., a component was considered fixed only if it proved irremovable with one hand during revision surgery [19]. In contrast, studies by Murer et al. and Hirschmann et al. stated that the potential of toggling the implant after standard approach (including synovial debridement and removal of osteophytes) should lead to the determination that the component is loose [9, 20].

This study is subject to several limitations, and as such, the interpretation of its findings should take into account the following observations. First, it is important to note the absence of a universally agreed‐upon framework for conducting Delphi consensus studies. Incorporating additional rounds or facilitating an open dialogue to discuss results, and providing nuanced statements and explanations, may have enhanced or expanded the consensus. However, this study proceeded according to a predefined design that aligns with widely accepted methodological standards [7, 11]. Second, despite the lead author's attempts to create a diverse international panel, the representation was not entirely comprehensive. The panel exhibited an overrepresentation of European (notably Dutch) and American members. This definition should be used solely as threshold for intraoperative observations as a reference test in diagnostic test studies. The clinical decision to revise a component should be based on a comprehensive assessment of the patient's condition, including clinical symptoms, imaging findings and other diagnostic criteria.

## CONCLUSION

There is high variability in factors contributing to the determination that a TKA or UKA component should be judged as loose, yet using this Delphi method consensus was reached on the visibility of fluid motion at the interface between the TKA component and/or cement and the bone observed during specific movements, such as flexion, extension, varus or valgus stress or when gently applying direct force with a finger or instrument, as definition for intraoperatively determined knee arthroplasty component loosening. This new definition should be used as a threshold diagnostic test studies where intraoperatively determining of component loosening is employed as reference test.

## Members of the International Consensus Panel

Claudia Arias, Johannes Beckmann, Lennard van den Boom, Bert Boonen, Benjamin V. Bloch, Nicolaas C. Budhiparama, Antonia F. Chen, Rüdiger von Eisenhart‐Rothe, Rutger van Geenen, Enrique Gómez‐Barrena, Heiko Graichen, James A. Harty, Roel Hendrick, Michael T. Hirschmann, Arthur J. Kievit, Lucien Keijser, Robin W. T. M. van Kempen, Jean‐Yves Jenny, Sébastien Lustig, Hermes H. Miozzari, Arun Mullaji, Sam I. S. Oussedik, Kailash Patil, Carsten Perka, Matthias U. Schafroth, Reha N. Tandogan, Emmanuel Thienpont, Peter C. M. Verdonk, Jonathan Vigdorchik, Daniel C. Wascher, Phil Walmsley, Jan Victor, Adolph Joseph Yates Jr and Simon van Laarhoven.

## AUTHOR CONTRIBUTIONS

**George S. Buijs**: Conceptualisation; data curation; formal analysis; investigation; methodology; project administration; resources; supervision; validation; visualisation; writing—original draught; writing—review and editing; given final approval. **Alex B. Walinga**: Methodology, project administration, formal analysis. **Arthur J. Kievit**, **Matthias U. Schafroth**, **Leendert Blankevoort** and **Michael T. Hirschmann**: Conceptualisation; methodology; supervision; writing—review and editing; given final approval.

## CONFLICTS OF INTEREST STATEMENT

Leendert Blankevoort, Arthur J. Kievit and Matthias T. Schafroth are listed as inventors on a patent for a loading device that can be used to quantify and visualise implant displacement. The remaining authors declare no conflict of interest.

## ETHICS STATEMENT

The authors have nothing to report.

## Data Availability

The data that support the findings of this study are available on request from the corresponding author.
